# Lung Cancer in Chinese Women: Evidence for an Interaction between Tobacco Smoking and Exposure to Inhalants in the Indoor Environment

**DOI:** 10.1289/ehp.0901587

**Published:** 2010-05-14

**Authors:** Li Tang, Wei-Yen Lim, Philip Eng, Swan Swan Leong, Tow Keang Lim, Alan W.K. Ng, Augustine Tee, Adeline Seow

**Affiliations:** 1 Department of Epidemiology and Public Health, National University of Singapore, Singapore; 2 Respiratory and Critical Care Medicine, Singapore General Hospital, Singapore; 3 Department of Medical Oncology, National Cancer Centre, Singapore; 4 Department of Medicine, National University Hospital, Singapore; 5 Department of Respiratory Medicine, Tan Tock Seng Hospital, Singapore; 6 Department of Respiratory Medicine, Changi General Hospital, Singapore

**Keywords:** Chinese, combustion sources, females, inhalants, interaction, lung cancer, tobacco smoking

## Abstract

**Background:**

Epidemiologic data suggest that Chinese women have a high incidence of lung cancer in relation to their smoking prevalence. In addition to active tobacco smoke exposure, other sources of fumes and airborne particles in the indoor environment, such as cooking and burning of incense and mosquito coils, have been considered potential risk factors for lung cancer.

**Objectives:**

We used a case–control study to explore effects of inhalants from combustion sources common in the domestic environment on lung cancer and their modification by active tobacco smoking.

**Methods:**

We analyzed 703 primary lung cancer cases and 1,578 controls. Data on demographic background and relevant exposures were obtained by face-to-face interviews in the hospital.

**Results:**

We observed a positive relationship with daily exposure to incense or mosquito coils and to cooking fumes only among smokers, and no association among lifetime nonsmokers. Interactions between smoking and frequency of cooking, or exposure to incense or mosquito coils were statistically significant and consistent with synergistic effects on lung cancer. The odds ratio (OR) comparing smokers without daily incense or mosquito coil exposure with nonsmokers without daily exposure was 2.80 [95% confidence interval (CI), 1.86–4.21], whereas the OR comparing smokers with daily exposure to the same referent group was 4.61 (95% CI, 3.41–6.24). In contrast, daily exposure to incense or mosquito coils was not associated with lung cancer among nonsmokers (OR = 0.91; 95% CI, 0.72–1.16). We observed the same pattern of associations for smokers without (OR = 2.31; 95% CI, 1.52–3.51) and with (OR = 4.50; 95% CI, 3.21–6.30) daily cooking exposure compared with nonsmokers, with no evidence of an association with daily cooking exposure among nonsmokers.

**Conclusion:**

Our results suggest that active tobacco smoking not only is an important risk factor for development of lung cancer, but also may cause smokers to be more susceptible to the risk-enhancing effects of other inhalants.

Lung cancer accounts for a substantial proportion of cancer incidence and mortality throughout the world (Parkin et al. 2005). In addition to tobacco smoke exposure (both active and secondhand), fumes and airborne particulates in the indoor environment have been considered as potential risk factors for lung cancer; examples include exposure to cooking oil fumes, cooking and heating fuels (household coal and wood combustion), incense and mosquito coils, and indoor radon (Ko et al. 2000; Wang et al. 2002; Yu et al. 2006; Zhang and Smith 2007).

Exposure to cooking fumes may potentially play a role in the occurrence of lung cancer. Cooking oil fumes are known to contain at least two carcinogenic compounds, benzo[*a*]pyrene and 2,4-decadienal, which induce lung cell survival and proliferation via the nuclear factor-κB pathway (Hung et al. 2005, 2007). Cumulative exposure to cooking (frequency and duration) by means of frying (stir-frying, frying, and deep-frying) was positively associated with the risk of lung cancer among female nonsmokers in Hong Kong (Yu et al. 2006). Women nonsmokers were at higher risk for lung cancer if they were exposed to cooking oil fumes emitted at high temperatures, and the risks were higher when the fumes were not reduced by an extractor (Ko et al. 2000).

The combustion by-products from heating and cooking are also sources of indoor air pollution. In Canada, a case–control study of lung cancer in 1996–2001 reported that, among women, the odds ratio (OR) for those exposed to both traditional heating and cooking sources (coal and wood) was 2.5 [95% confidence interval (CI), 1.5–3.6] relative to women not exposed to either source (Ramanakumar et al. 2007). Traditional heating and cooking fuels (coal and wood) produce a variety of indoor pollutants, including respirable particles, heavy metals, polycyclic aromatic hydrocarbons (PAHs), carbon monoxide, carbon dioxide, nitrogen dioxide, sulfur dioxide, and formaldehyde (Zhang and Smith 2007). The use of coal for heating has been implicated in the high incidence of lung cancer among residents of Xuanwei, China (Lan et al. 2002).

Incense burning, a traditional practice in Chinese households, is also powerful producer of particulate matter, and incense smoke contains carcinogens such as PAHs, carbonyls, and benzene (Lin and Tang 1994; Lofroth et al. 1991). Incense smoke condensates have mutagenic and genotoxic activities, and the genotoxicity of certain incense smoke condensates in mammalian cells has been shown to be higher than that of tobacco smoke condensate (Chen and Lee 1996; Rasmussen 1987). The potential impact of incense on health has also been studied outside the home (Chiang and Liao 2006; Chiang et al. 2009). A large prospective cohort study in Singapore reported an association between long-term incense use and the development of squamous cell carcinomas of the respiratory tract, particularly among women (Friborg et al. 2008).

Mosquito coils are frequently burned indoors in Asia and to a limited extent in other parts of the world, including the United States (World Health Organization 1998). The major ingredients of the mosquito coils are pyrethrins and plant-based materials, such as wood powder, coconut shell powder, and joss powder, as well as binders, dyes, oxidants, and other additives to allow for controlled smoldering (Chen et al. 2008; Krieger et al. 2003). The combustion of these materials generates large amounts of submicrometer particles and gaseous pollutants. These submicrometer particles may reach the lower respiratory tract and could be coated with a wide range of organic compounds, such as PAHs. A study of mosquito coil smoke and lung cancer in Taiwan between 2002 and 2004 showed that lung cancer risk among smokers with the highest exposure to mosquito coil smoke was 14 times higher than nonsmokers without this exposure (Chen et al. 2008).

In this study, we used the case–control study design to investigate whether inhalant exposure from these sources plays a significant role in enhancing risk of lung cancer among Singapore Chinese women, a population with a large proportion of nonsmokers. We also wished to explore whether the impact of these compounds is modified by active tobacco smoke exposure.

## Materials and Methods

Participants were cases and controls who were recruited for two hospital-based case–control studies during 1996–1998 and 2005–2008, from the five major public hospitals in Singapore. Both studies used similar methods and questionnaires. Eligible cases were Chinese females with newly diagnosed primary carcinoma of the lung. The average time between diagnosis and interview was 22 days (79.4% were interviewed within 1 month of diagnosis). A total of 703 lung cancer patients (89.2% of those identified as eligible) agreed to participate. Histologic or cytologic reports were reviewed and confirmed the diagnosis of primary lung carcinoma in 674 cases; 29 cases were confirmed on the basis of radiologic investigations, in which metastatic cancer to the lung from other sites was deemed to be unlikely on clinical grounds. Controls were selected from Chinese female patients, frequency matched for age (within 5 years), hospital admitted to, and date of admission (within 1 month). Patients admitted for a diagnosis and treatment of cancer or chronic respiratory disease were excluded, and no more than 10% of controls were recruited within a single diagnostic category.

The response rate among controls was 90.6%, and data from a total of 1,578 controls were available for analysis. Control patients were admitted for a wide range of conditions: 27% had diseases of skin, bones, joints, and connective tissue; 11% were admitted for gastrointestinal or hepatobiliary system complaints; 14% were admitted for acute trauma; 8% were admitted for neurological or psychiatric conditions; and 12% had diseases of the cardiovascular system.

Both eligible cases and controls gave written, informed consent for the interview and the tracing of their medical records, and the study was approved by the Institutional Review Board of the National University of Singapore and the participating health care institutions.

All subjects were interviewed in person by trained interviewers, using a structured questionnaire. Interviewers were not blinded to case or control status, but we recorded and reviewed at random a sample of interviews conducted to ensure standardization of the data collection processes. The structured questionnaire covered demographic characteristics, occupational history, active smoking history, family history of cancer, personal medical history (e.g., history of tuberculosis), dietary intake of fruits and vegetables, and indoor environmental exposures (including secondhand smoking exposure, cooking exposure, and exposure to incense and mosquito coil burning).

The participant’s smoking history included the number of cigarettes smoked daily and the total duration of smoking. A regular smoker was defined as one who smoked at least one cigarette per day for ≥ 1 year. Ex-smokers were smokers who had stopped smoking for ≥ 30 days at the time of interview. Questions on secondhand smoke exposure included “Did any of your household members smoke (including spouse, parents, children, or any other relative/friend living with you) in your presence more than once a week?” Family history of cancer was defined as the presence of any cancer within first-degree relatives. Information on intake of fruits and vegetables was collected using a semiquantitative food frequency questionnaire that elicited the usual weekly number of servings of 17 fruit and 21 vegetable items over the 3 years before admission. The time period for inhalant exposure was set at 25 years before admission, and all questions asked participants to recall exposures 25 years before age of diagnosis of lung cancer (or age at admission for controls). For cooking exposure, participants were asked about the frequency with which they personally cooked at home (with six categories of response, ranging from “never” to “more than once a day”), the cooking methods used, and the age at which they began to do this regularly. The frequency of incense/mosquito coil burning (i.e., less than daily, once daily, more than once a day/throughout the day, throughout the day and night) was also ascertained. In each case, the question was asked (e.g., “How often were joss sticks, scented coil/powder burnt inside your house?”), and the respondent asked to select the most appropriate frequency category. For the purpose of the analysis, exposures were categorized as less than daily (“< daily”) and once or more every day (“daily”).

ORs and their 95% CIs were calculated for risk of lung cancer for smokers and nonsmokers separately using unconditional logistic regression adjusting for age (years), education (years), housing type, secondhand smoke exposure (daily vs. less than daily exposure), history of cancer in the first-degree relative, duration of smoking (in years; for ex-smokers and current smokers), fruit and vegetable intake (servings/week), and study set (1996–1998 or 2005–2008 study). These adjustment variables were modeled with age, fruit consumption, and vegetable consumption as continuous variables, and all other variables as categorical ones, with the respective categories, as shown in Table 1. Among smokers, intensity of smoking was highly correlated with duration, and further adjustment for the former did not affect the ORs, so it was excluded in the final statistical model. We used STATA statistical software (version SE 10.1; StataCorp LP, College Station, TX, USA) for data analyses. All *p*-values were calculated using two-tailed statistical tests, and the criterion for significance was set at *p* < 0.05. Interactions were assessed using the likelihood ratio test to estimate *p*-values; in each test for interaction, models that included the interaction term were compared with those that did not.

## Results

We analyzed data from 703 cases with primary lung cancer and 1,578 controls. Data on exposures of interest and potential confounders were available for almost all participants, with the highest proportion of missing data for exposures being 1.1% (for the cooking variable). Table 1 describes sociodemographic characteristics of the cases and controls. Cases were significantly more likely to be current smokers (17.9% vs. 5.6%) or ever smokers (20.3% vs. 7.3%; ex-smokers: age-adjusted OR = 3.85; 95% CI, 2.93–5.01; current smokers: age-adjusted OR = 4.49; 95% CI, 3.34–6.02). They were also more likely to have been exposed to secondhand smoke at home daily (51.9% vs. 45.9% for controls). Cases had a higher proportion of family history of cancer than did controls (24.3% vs. 18.5%). The mean weekly number of servings of fruits and vegetables was lower among cases than among controls (6.8 and 21.4 vs. 9.0 and 25.6, respectively).

Associations between lung cancer and exposure to incense or mosquito coils, and with exposure to daily cooking, were strongly dependent on smoking status; Table 2 presents these results separately for smokers and nonsmokers. We observed a statistically significant positive relationship only among smokers and observed no association among lifetime nonsmokers. Among smokers, women who cooked daily had a higher risk than those who cooked less than daily (adjusted OR = 1.61; 95% CI, 1.01–2.56). Also, smokers with exposure to incense or mosquito coils daily were more likely to have lung cancer than those with less frequent exposure (OR = 1.53; 95% CI, 0.97–2.41) after adjustment for potential confounders. Daily use of charcoal or wood stove was not associated with lung cancer in either smokers or nonsmokers.

We found a statistically significant interaction between smoking and exposure to incense or mosquito coils (*p* = 0.016) or frequency of cooking (*p* < 0.001), respectively, after adjustment for potential confounders (Table 3). The OR comparing smokers without daily incense or mosquito coils exposure with nonsmokers without daily exposure was 2.80 (95% CI, 1.86–4.21), whereas the OR comparing smokers with daily exposure with the same referent group was 4.61 (95% CI, 3.41–6.24). In contrast, daily exposure to incense or mosquito coils was not associated with lung cancer among nonsmokers (OR = 0.91; 95% CI, 0.72–1.16). We observed the same pattern of associations for smokers without (OR = 2.31; 95% CI, 1.52–3.51) and with daily cooking exposure (OR = 4.50; 95% CI, 3.21–6.30) compared with nonsmokers, with no evidence of an association with daily cooking exposure among nonsmokers. We observed the same pattern for wood stove use, although the interaction was not statistically significant (*p* = 0.061). We found no interaction between smoking and daily use of charcoal (*p* = 0.128).

## Discussion

We examined the effects of cooking and exposure to burning of incense and mosquito coils on lung cancer risk among Singapore Chinese women, and their modification by active tobacco smoking exposure. We observed strong interactions between exposure to these sources and smoking on lung cancer risk. The results indicate that active tobacco smoking not only is an important risk factor for development of lung cancer, but also may cause smokers to be more susceptible than nonsmokers to adverse effects of these inhalants on lung cancer as well.

A possible explanation for our findings is the presence of a chronic inflammatory state in the airways induced by smoking. Tobacco smoke carcinogens are known to activate proinflammatory responses through the action of prooxidative chemicals, leading to the release of cytokines, production of reactive oxygen species (ROS), and ultimately DNA damage (Azad et al. 2008; Hecht 2008). A chronic inflammatory process in the lung could also lead directly to DNA damage, enhance the effects of other carcinogenic exposures, and stimulate cell proliferation and growth (Ohshima and Bartsch 1994). Burning incense generates high concentrations of ROS in the particulate gas phase of the emissions, and might damage DNA and other biomolecules when inhaled (Szeto et al. 2009).

Our findings that these exposures are not associated with risk among nonsmokers are at variance with other studies that reported positive associations in nonsmokers. The OR for female nonsmokers cooking three meals/day compared with those cooking one meal/day was 3.4 (95% CI, 1.6–7.0) in a study conducted in Taiwan (Ko et al. 2000). In the study among women in Taiwan, higher frequency of mosquito coil smoke use was positively associated with lung cancer in both smokers and nonsmokers, although the interaction with cigarette smoke was synergistic (Chen et al. 2008), as in the present study. Differences in cooking practices, use of fume extractors, type and intensity of use of mosquito coils, or simply in the average amount of time spent at home may contribute to the difference in findings among studies, even within Chinese populations. The proportion of women who had never been employed outside the home in our study was only 22% among controls, suggesting that overall exposure to air pollutants in the domestic environment may be less substantial in our population than in more traditional societies.

Contrary to previous reports, we did not find a significant association between use of charcoal or wood stoves and lung cancer risk, among either smokers or nonsmokers. In Singapore, local residents infrequently use traditional fuels (charcoal or wood) and usually use modern fuels (gas, kerosene, or electricity) for cooking, and the low frequency of use may be the chief explanation for our findings. We also recognize that there are limitations to the data presented. Because the study is retrospective, recall and reporting biases by subjects are inevitable concerns. We believe that these biases are not likely to be differential, because we did not make our hypothesis known to our participants, and the possible association between inhalant exposure and lung cancer is not widely known among the public. These errors, if present, would probably shift the association toward the null, because they would likely affect both cases and controls to the same extent.

## Conclusions

Our study suggests that active tobacco smoking not only is an important risk factor for development of lung cancer, but also may cause smokers to be more susceptible to the risk-enhancing effects of exposure to cooking and burning of incense and mosquito coils. A possible mechanism consistent with recent findings is the presence of a chronic inflammatory state in the airways induced by smoking. The interaction observed supports a model in which host susceptibility acts in concert with the exposures of interest to promote lung carcinogenesis. On the other hand, we found no evidence that these specific exposures contribute to increased risk of lung cancer among nonsmokers. Because cooking and burning of incense and mosquito coils are fairly common exposures in the indoor environment, it is important that smokers be aware of the significant additional risk afforded by these exposures. Although our results suggest a weaker effect, if any, among nonsmokers, further research is needed to establish more definitively the level of risk from these ubiquitous compounds in the domestic environment.

## Figures and Tables

**Table 1:** Sociodemographic characteristics of lung cancer cases and controls, Singapore Chinese women [*n* (%)].

Characteristic	Cases (*n* = 703)	Controls (*n* = 1,578)	*p*-Value^a^
Age [years (mean ± SD)]^b^	65.9 ± 11.9	64.1 ± 12.3	0.001

Birthplace			0.001
Singapore	443 (63.0)	1031 (65.3)	
Malaysia	92 (13.1)	271 (17.2)	
China	145 (20.6)	234 (14.8)	
Other	23 (3.3)	42 (2.7)	

Education (years)			0.037
None	342 (48.7)	678 (43.0)	
≤ 6	193 (27.5)	491 (31.1)	
≥ 7	167 (23.8)	409 (25.9)	

Dwelling			0.020
Flat, 1–3 rooms	255 (36.5)	613 (39.0)	
Flat, ≥ 4 rooms	332 (47.6)	775 (49.3)	
Private apartment or house	111 (15.9)	183 (11.7)	

Marital status			0.956
Ever married	654 (93.0)	1,467 (93.0)	
Never married	49 (7.0)	111 (7.0)	

Occupational status			0.018
Currently employed outside home	154 (21.9)	415 (26.3)	
Ever employed outside home	360 (51.2)	809 (51.3)	
Never employed outside home	189 (26.9)	352 (22.3)	

Smoking history			< 0.001
Nonsmoker	434 (61.7)	1,375 (87.1)	
Ex-smoker^c^	143 (20.3)	115 (7.3)	
Current smoker	126 (17.9)	88 (5.6)	

Secondhand smoke exposure at home			0.009
< Daily	335 (48.1)	848 (54.1)	
Daily	361 (51.9)	720 (45.9)	

Family history of cancer^d^			0.001
No	532 (75.7)	1,286 (81.5)	
Yes	171 (24.3)	292 (18.5)	

Servings/week of fruit (mean ± SD)	6.8 ± 8.5	9.0 ± 8.6	< 0.001

Servings/week of vegetables (mean ± SD)	21.4 ± 19.3	25.6 ± 21.3	< 0.001

a Pearson chi-square test for categorical variables and *t*-test for continuous variables.

b Age at diagnosis (cases) and age at interview (controls).

c Had not smoked any cigarette in the 30 days before admission.

d First-degree relative with history of cancer of any site.

**Table 2:** Adjusted ORs^a^ and 95% CIs for lung cancer by cooking, incense or mosquito coil use, and charcoal and wood stove use, by smoking status.

	Current or ex-smokers		Nonsmokers	
Exposure factor	Cases/controls	OR (95% CI)	Cases/controls	OR (95% CI)
Cooking frequency				
< Daily	58/73	1.00	145/385	1.00
Daily	210/130	1.61 (1.01–2.56)	282/972	0.89 (0.68–1.16)

Use of incense or mosquito coils				
< Daily	62/65	1.00	169/488	1.00
Daily	207/138	1.53 (0.97–2.41)	265/887	0.90 (0.71–1.14)

Use of charcoal stove				
< Daily	239/180	1.00	406/1,236	1.00
Daily	30/21	1.08 (0.55–2.12)	26/129	0.67 (0.43–1.05)

Use of wood stove				
< Daily	215/167	1.00	387/1,177	1.00
Daily	54/33	1.25 (0.74–2.12)	45/193	0.81 (0.56–1.17)

a Adjusted as described in “Materials and Methods.”

**Table 3:** Combined effect estimates for lung cancer in association with indoor inhalants and smoking.

Exposure group	Smoking status	*n*	OR (95% CI)^a^
Cooking frequency			
< Daily	Nonsmokers	530	1.00
Daily	Nonsmokers	1,254	0.83 (0.64–1.08)
< Daily	Current or ex-smokers	131	2.31 (1.52–3.51)
Daily	Current or ex-smokers	340	4.50 (3.21–6.30)
*p*-Value (interaction)^b^ < 0.001			

Use of incense or mosquito coils			
< Daily	Nonsmokers	657	1.00
Daily	Nonsmokers	1,152	0.91 (0.72–1.16)
< Daily	Current or ex-smokers	127	2.80 (1.86–4.21)
Daily	Current or ex-smokers	345	4.61 (3.41–6.24)
*p*-Value (interaction)^b^ = 0.016			

Use of charcoal stove			
< Daily	Nonsmokers	1,642	1.00
Daily	Nonsmokers	155	0.67 (0.43–1.04)
< Daily	Current or ex-smokers	419	4.08 (3.21–5.18)
Daily	Current or ex-smokers	51	4.88 (2.68–8.91)
*p*-Value (interaction)^b^ = 0.128			

Use of wood stove			
< Daily	Nonsmokers	1,564	1.00
Daily	Nonsmokers	238	0.78 (0.55–1.13)
< Daily	Current or ex-smokers	382	3.95 (3.08–5.07)
Daily	Current or ex-smokers	87	5.48 (3.42–8.79)
*p*-Value (interaction)^b^ = 0.061			

a Adjusted as described in “Materials and Methods.”

b *p*-Value for the likelihood ratio test for interaction between smoking and cooking, incense or mosquito coils, charcoal stove, and wood stove.
